# Supplementary material for: Kudoa septempunctata Spores Cause Acute Gastroenteric Symptoms in Mouse and Musk Shrew Models as Evidenced In Vitro in Human Colon Cells
Source: Pathogens. 2023 May 20;12(5):739. doi: 10.3390/pathogens12050739 (PMC10220943; doi:10.3390/pathogens12050739)
Supplement: Supplementary file 1 [file pathogens-12-00739-s001.zip › pathogens-2378798-supplementary.pdf]

Supplementary Material

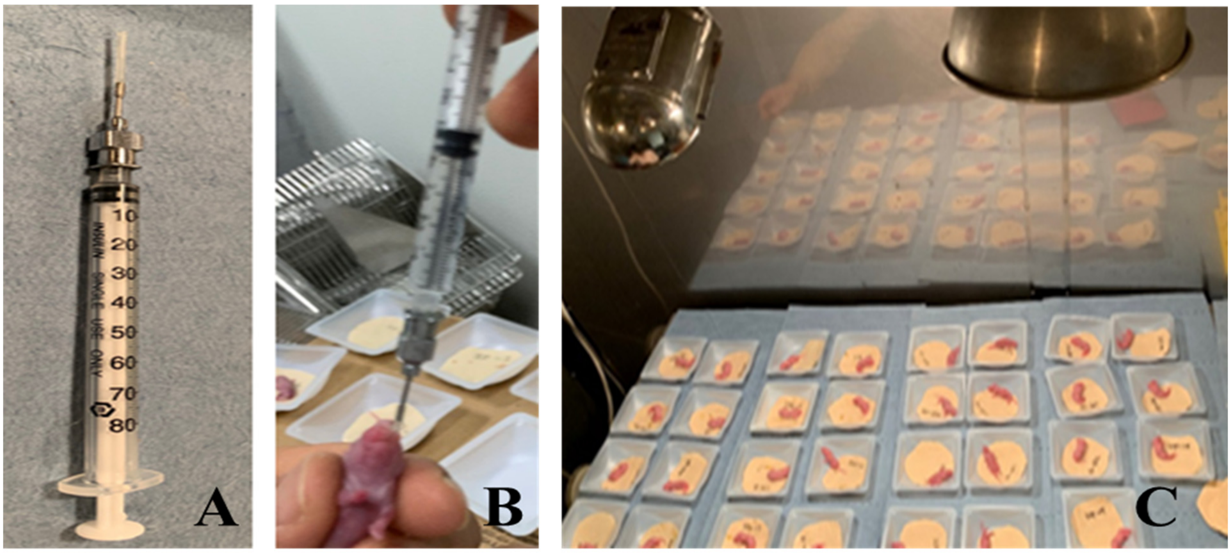

Figure S1. Administration of *Kudoa septempunctata* spores and observations in suckling mice. (**A**) A 1-mL syringe and needle capped with a polyethylene tube. (**B**) Spores were administered to suckling mice using a syringe. (**C**) Suckled mice were arrayed under two incandescent lamps. All suckling mice were handled throughout the experiment, received from a single delivery, stabilized for 1 h, administered with the spores, and observed for diarrhea in a walk-in-incubator maintained at 27 °C and 50% humidity.

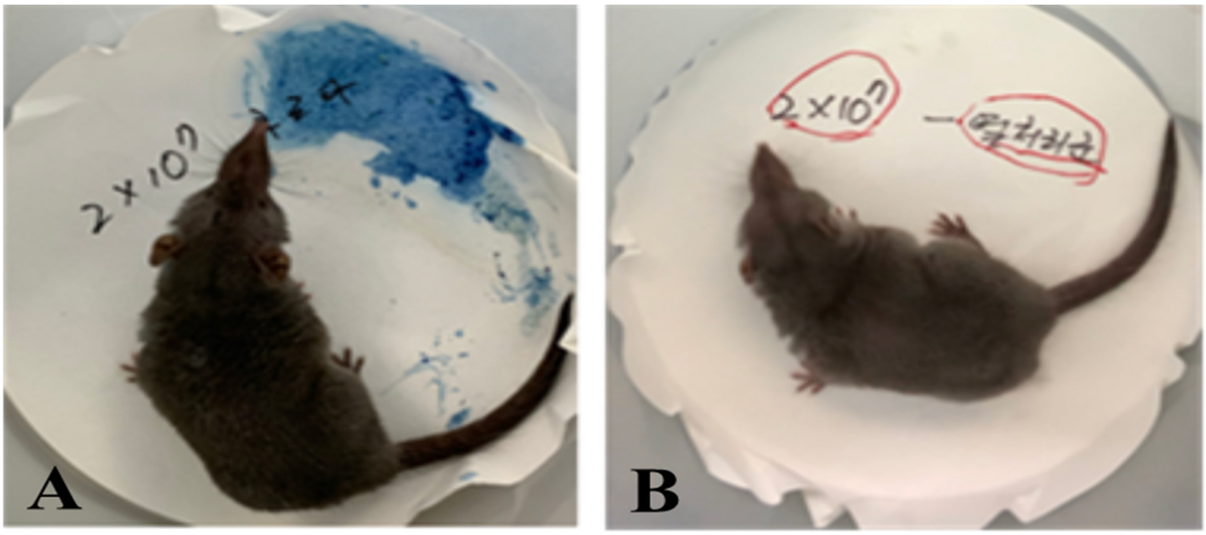

Figure S2. House musk shrews administered with 2×10^7 *Kudoa septempunctata* spores. (**A**) Vomitus from live spores administered to shrews. (**B**) Control shrew with heat-inactivated spores.
